# Syphilis in the Inferior Animals

**Published:** 1872-05-01

**Authors:** E. Andrews

**Affiliations:** Professor of Principles and Practice of Surgery in Chicago Medical College


### SYPHILIS IN THE INFERIOR ANIMALS.

BY E. ANDREWS, M.D.,

(*Professor of Principles and Practice of Surgery in Chicago Medical College*).

The distinguished advocate of syphilization in Paris, Dr. Anzias Turenne, proved, as he supposed, by inoculations, that while the inferior animals could have soft chancre, they were incapable of constitutional syphilis. This conclusion, though resting solely on the narrow basis of one man's experiments, appears to be accepted by all the eminent syphilographers of Europe; and being the prevailing doctrine, I have myself often quoted it as true.

On consulting a number of leading works on Veterinary Surgery, I find no allusion to syphilis except in Gamge, who doubtfully speaks of certain primary sores, but of no secondary in inferior animals. A veterinary surgeon of this city, however, informs me that he has met three cases of what he considers well marked constitutional syphilis in breeding animals.

The first case was that of a bull in England. The first symptom was a chancre on the penis, accompanied with inflammation and swelling, and followed by secondary eruptions on the skin.

Case second was on a celebrated imported bull. The first symptom was a discharge from the prepuce, with swelling and inflammation. This was followed, first by moist ulcers in the mouth, and later by eruptions with large scabs on the skin.

Case third was that of a boar which was supposed to have a primary sore, which was followed by eruptions on the skin. The veterinary surgeon above mentioned says that gonorrhœa is very common in bulls.

These three cases are not enough to bring us to a decisive result, but they show that the subject needs a re-investigation. Domestic animals are certainly less subject to syphilis than men; but they may, after all, have it in a mild and modified form. If experiments should prove the latter hypothesis, the question would immediately arise whether this modified form could be used, like vaccinia, as a prophylactic against syphilis proper. Who will make the experiment?

